# Subglacial hydrology and the formation of ice streams

**DOI:** 10.1098/rspa.2013.0494

**Published:** 2014-01-08

**Authors:** T. M Kyrke-Smith, R. F Katz, A. C Fowler

**Affiliations:** 1 Department of Earth Sciences, University of Oxford, Oxford, UK; 2 OCIAM, University of Oxford, Oxford, UK; 3 MACSI, University of Limerick, Limerick, Ireland

**Keywords:** ice sheets, ice streams, subglacial hydrology, hydraulic runaway

## Abstract

Antarctic ice streams are associated with pressurized subglacial meltwater but the role this water plays in the dynamics of the streams is not known. To address this, we present a model of subglacial water flow below ice sheets, and particularly below ice streams. The base-level flow is fed by subglacial melting and is presumed to take the form of a rough-bedded film, in which the ice is supported by larger clasts, but there is a millimetric water film which submerges the smaller particles. A model for the film is given by two coupled partial differential equations, representing mass conservation of water and ice closure. We assume that there is no sediment transport and solve for water film depth and effective pressure. This is coupled to a vertically integrated, higher order model for ice-sheet dynamics. If there is a sufficiently small amount of meltwater produced (e.g. if ice flux is low), the distributed film and ice sheet are stable, whereas for larger amounts of melt the ice–water system can become unstable, and ice streams form spontaneously as a consequence. We show that this can be explained in terms of a multi-valued sliding law, which arises from a simplified, one-dimensional analysis of the coupled model.

## 1. Introduction

Variations in sliding velocity at the ice–bed interface can cause extreme spatial and temporal differentials in flow speed throughout an ice sheet. In particular, while there are many locations in Antarctica where the ice has zero velocity, velocities of up to thousands of metres per year are observed in some coastal areas [1]. In the fast-flowing regions, the rapid basal sliding is attributed to the presence of meltwater at the bed [2]. Frictional resistance at the bed is intrinsically linked to the water pressure in the meltwater drainage system [3–6]. This effect is best considered in terms of the effective pressure (the difference between ice overburden pressure and water pressure); sliding at the bed can occur when the effective pressure is small and the weight of the ice is largely supported by the underlying water. The dependence of the basal sliding velocity on both the basal shear stress and the effective pressure at the bed is typically modelled with a sliding law [7–9]. To improve our understanding of variations in basal velocity, we must investigate the physical basis and consequences of the sliding law.

It is the behaviour of the subglacial hydrological system that governs the effective pressure at the ice–bed interface. There are two contrasting types of subglacial drainage systems beneath ice sheets that are commonly discussed in the literature. If there is little meltwater production, the bed can be effectively drained by a distributed system of cavities [10,11] or through thin, patchy films [12]. With this style of drainage, increasing meltwater production increases the water pressure beneath the ice, which lowers the effective pressure, and hence results in more rapid sliding [10,13–15]. By contrast, subglacial water transport may occur through larger scale Röthlisberger channels. These provide efficient drainage for meltwater, resulting in low water pressure at the bed, corresponding to high effective pressure, and hence slower sliding velocities [16]. Beneath Antarctica, however, there is more evidence of high water pressure, distributed drainage systems [17].

In this work, we are particularly interested in ice streams and their subglacial hydrology. A significant proportion of all ice discharge occurs through ice streams, despite the fact that they are only found over a small portion of an ice sheet. In Antarctica, for example, it is estimated that ice streams cover approximately 10% of the ice-sheet’s surface, but transmit up to 90% of drainage [1,18]. There is evidence suggesting the presence of an active subglacial water system in West Antarctica [19] and it is therefore important to understand the effect of this on the ice flow. Previous work has shown that multi-valued sliding laws can result in ice-stream flow [11,13,20–23]. More specifically, Fowler & Johnson [24,25] demonstrated that a ‘hydraulic runaway’ mechanism is suggestive of a triple-valued sliding law, based on thermomechanical feedbacks that arise as a result of having a distributed drainage system at the bed. Sayag & Tziperman [26] invoked a switch in drainage system as the motivation for a triple-valued sliding law, suggesting that each of two stable branches correspond to different drainage patterns at the bed.

While implementing a multi-valued flux law as a boundary condition may produce ice-stream behaviour, using knowledge of the subglacial drainage system beneath Antarctica more directly to explain ice-stream emergence would further our understanding of the system. Evidence from Antarctica suggests that the rapid basal velocities in ice streams are enabled by the presence of a layer of till at the base of the sheet [27–30]. For likely permeabilities and till thickness, Darcian porous flow through the sediment would be too slow to evacuate all the meltwater present at the bed [31,32] and the till is therefore water saturated and is thought to deform with Coulomb-plastic behaviour [33–37]. Knowledge of the yield strength of the till is important, but more information than this is required about the hydrological system and how its evolution affects the ice above. Recent work looking at ice-stream formation over till either disregards water transport altogether [38] or uses a simple diffusion equation to describe the evolution of a water layer over saturated till, assuming the water ‘diffuses’ from high to low effective thickness [39–41]. In this work, we wish to develop a more physically motivated model for the water layer and investigate the potential link between the hydrological system and multi-valued flux laws. This will extend the work done by Fowler & Johnson [24,25], who introduced the concept of hydraulic runaway and considered the feedbacks in a simplified one-dimensional model. In our more detailed hydrological model, we consider meltwater flowing over saturated, relatively impermeable till. A water film exists only if it is shallow enough not to submerge all surrounding bed protrusions [42]. If the water depth increases beyond some critical value (that at which all surrounding clasts are drowned by the water), the water film becomes unstable to the formation of local water streams incised in the sediment [32]. There is uncertainty surrounding some of the parametrizations made in the formulation of the model; the purpose of this work is to investigate the qualitative implications and feedbacks that arise from the coupling of the meltwater and ice.

The paper is organized as follows. In §2, we present the ice-sheet model and derive governing equations for the water film depth and the effective pressure at the bed, before non-dimensionalizing the model in §3. Section 4 considers behaviour of the water layer itself in more detail, specifically considering what happens in the model once the water layer becomes deep enough to submerge all surrounding clasts. In §5, we present numerical solutions to the fully coupled ice-water model, which, under certain conditions, results in ice-stream flow, and we discuss how this can be explained in relation to a multi-valued flux law and the chosen parametrizations in §6.

## 2. Governing equations

### (a) Ice flow

Given that ice is an incompressible, viscous fluid, an ice sheet moving under gravity is accurately described by Stokes flow. The low aspect ratio of ice sheets (approx. 10^−3^) allows simplifications to be made to the three-dimensional nonlinear equations, with two end-member models being the shallow ice approximation (SIA) and the shallow shelf approximation (SSA). The former is a classical lubrication approximation, valid for flow frozen to the bed, and so dominated by vertical shear stresses [43–45]. The SSA, on the other hand, does not allow for internal deformation, and so provides an accurate flow description where horizontal ice velocities do not vary with depth [46,47], for example in ice streams or on ice shelves.

In this work, we use a model that is a vertically integrated hybrid of the SIA and SSA. It takes into account both vertical shear stresses and membrane stresses, so providing a valid flow description for all flow regimes within a shallow ice sheet. This is particularly important when modelling the emergence of ice streams. The force balance includes basal stresses, driving stresses and membrane stresses. A complete description of the model is presented in previous work [48], along with a comparison between this model and similar higher order approaches [49,50]; here just the non-dimensional equations are outlined at the end of §3.

### (b) Subglacial water flow

**Figure 1.**
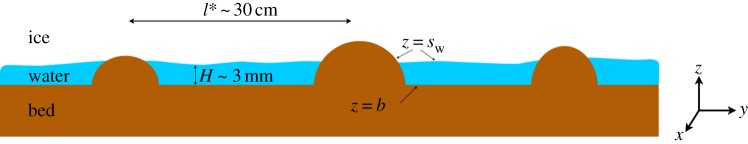
Geometry of the subglacial flow under consideration.

We conceive of water flowing at the base of an ice stream as illustrated in figure 1. The ice is underlain by sediments that are saturated by water. Typically, the water is at high pressure and the till is deformable. We presume that the till is not very permeable and expect that the water generated at the base might flow in some sort of distributed system. The simplest such flow is a thin film at the interface between the ice and the till. With a clean separation between the ice and the bed, as imagined by Weertman [51], the resulting flow is an unstable configuration [52]. However, we can suppose such a film could exist stably if it is thinner than the supporting clast size [42], which is reasonable if the water film thickness is of the order of millimetres. Of course, this film is still subject to instability if it grows deep enough to separate the ice and bed, and we can expect local-scale water streams to form once the water layer depth reaches some critical value at which surrounding bed protrusions are submerged [32]. Note that these water streams form over much smaller length scales than ice streams; in this work, we consider the larger scale averaged behaviour of these small-scale features at the bed and postpone the study of a more detailed process of the formation of water streams for future work.

We consider Cartesian axes (*x*,*y*,*z*) where *x* points in the ice flow direction, *y* is across-flow and *z* is vertically upwards. As shown in figure 1, the till surface is denoted by *z*=*b*, the lower ice surface by *z*=*s*_w_ and the resulting depth of the water film is
2.1
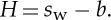

The upper ice surface is denoted by *z*=*s*_i_. The hydraulic head driving water flow at the ice–till interface is
2.2
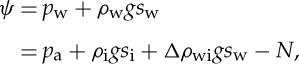

where *p*_w_ is water pressure, *p*_a_ is atmospheric pressure, *ρ*_w_ and *ρ*_i_ are the densities of water and ice, respectively, and *g* is gravitational acceleration. *Δρ*_wi_=*ρ*_w_−*ρ*_i_ and *N*=*p*_i_−*p*_w_ is the effective pressure. *p*_i_=*p*_a_+*ρ*_i_*g*(*s*_i_−*s*_w_) is the basal ice pressure. Note that (2.2) reflects the well-known fact that basal water flow is driven primarily by the ice surface slope, and the basal slope only contributes approximately 1/10 to the flow direction.

Given that the water flows in a film of depth *H*, mass conservation of water takes the form
2.3
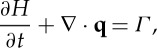

where *Γ* is the melt rate of the ice (with units kg m^−2^ s^−1^). It includes contributions from local heat sources as well as from frictional water dissipation in the subglacial water flow. More specifically,
2.4
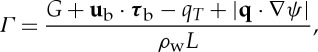

where *G* is the geothermal heat flux, **u**_b_ is the basal ice velocity, ***τ***_b_ is the basal ice stress, *q*_*T*_ is the sensible heat flux into the overlying ice and *L* is the latent heat. It is important to note that the frictional heat source **u**_b_⋅***τ***_b_ arises from an integration of the ice viscous dissipation term over the basal sliding region, and ***τ***_b_ and **u**_b_ here refer to conditions near the base of the far-field ice-sheet flow, but far from the actual interface [53]; they are the quantities used in the ice-sheet sliding law. At this stage, we are also neglecting dissipation in the ice owing to lateral shear—an effect that could be significant in the regions around ice-stream shear margins [54,55].

Assuming a local Poiseuille flow in the water film implies that
2.5
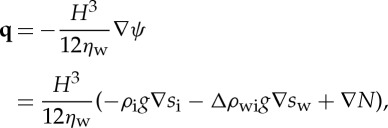

where *η*_w_ is the viscosity of water.

### (c) Ice closure relation

While mass conservation of water tells us how the water moves in response to a hydraulic potential gradient, we also require an equation to describe the evolution of the subglacial hydraulic system. By analogy with the closure equation of [16], we consider a balance between the opening and closure rates of the system; we take
2.6
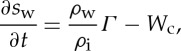

where the melt rate, *Γ*, is given by (2.4). The water film thickens as melt is produced and thins by closure owing to the excess ice overburden pressure. In the present situation, we conceive of the ice as being supported by load-bearing clasts, and *W*_c_ represents the viscous creep of ice around these supporting clasts. Under the assumption that clasts have spacing l* (figure 1), we take the rate of closure owing to viscous creep as [42]
2.7
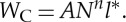

*A* is the rate factor and *n* the exponent from Glen’s flow law, which describes the rheological behaviour of ice [8]. *N* is the effective pressure; as the difference between ice overburden pressure and water pressure increases, the closure rate will also increase. Finally, the quantity l* is analogous to the channel width in the closure equation of Röthlisberger [16], assuming a single (wide) channel. Moreover, as the film thickness increases, the spacing between protruding clasts will increase as more of them become submerged; l* will therefore be an increasing function of *H*.

**Figure 2.**
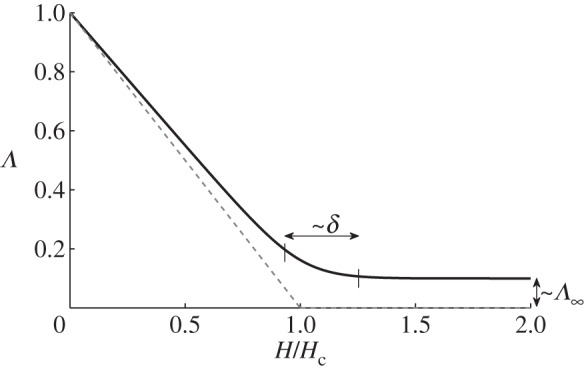
Plots of two choices for *Λ*(*H*). The dashed grey line is given by (2.9) and the solid black line by (2.11) with *δ*=0.1, 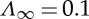.

There is little to constrain our choice for the functional dependence of l*(*H*). We assume that there is some film depth *H*_c_ at which all the clasts would become submerged and the ice would become locally clear of the bed. To be more specific, we will define
2.8
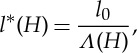

where *l*_0_ is a length scale that represents the typical clast spacing in the absence of water, and will be chosen to be consistent with observed effective pressures on the Whillans ice stream (B). We are then left to choose a function for *Λ*(*H*). The most simple choice, for illustrative purposes, would be a function such as
2.9
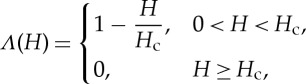

as illustrated by the dashed grey line in figure 2. When *H* reaches *H*_c_, the ice separates from the bed. From (2.6) with (2.7) and (2.8), we have that
2.10
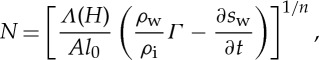

and it is clear that, with the above choice of *Λ*(*H*), *N*=0 when *H*=*H*_c_, implying that once the ice separates from the bed there is no basal stress. In this context, there is then no reason that *H* cannot increase further locally, with the effective pressure remaining zero. However, in practice, a sheet flow with depth *H*>*H*_c_ is an unstable configuration [52], as discussed above, and a water layer cannot increase indefinitely in depth. Furthermore, we do not expect that *N*=0 under ice streams; this would only be the case if there were a subglacial lake. We address this model issue by noting that the subglacial flow model has been formulated on the small scale; locally, areas of the bed may become submerged but we expect that on the larger scale there will still be areas of the bed providing support to the ice. More specifically, we presume that, when *H* approaches *H*_c_, the Walder instability is no longer suppressed, and the system becomes unstable to the formation of some kind of locally channelized drainage system at the ice–till interface [32]. We therefore consider the case where *Λ*(*H*) decreases to some small limit as *H* approaches *H*_c_, and specifically we take *Λ*(*H*) to be a function of the form
2.11
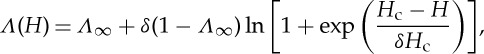

where both 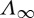 and *δ* are small. The solid black line in figure 2 illustrates this, with 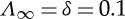. From (2.10), we have that *N*∼*Λ*^1/*n*^. This finite limit of *Λ*(*H*) therefore now means that the effective pressure does not reach zero in our model. Locally, we expect that there are small areas where the effective pressure is zero and *H*>*H*_c_, but on the larger scale (over which we are interested in modelling when investigating ice streams) there are regions of finite support, now on a length scale of several metres. Our choice of *Λ*(*H*) presented here is meant to represent this globally averaged behaviour in the simplest possible way.

It is also worth noting that, as we are considering water flow over sediment rather than hard bedrock, there should also be a description of bed evolution in the model. This would require a second closure relation for the bed elevation *b*, based on an Exner equation incorporating sediment transport. In the present work, however, we assume that there is no sediment transport so that *b* is fixed and *H*=*s*_w_−*b*. We therefore require only one closure equation. This is not an unreasonable assumption so long as there is no net change in bed elevation. There can still be some transport of sediment by virtue of the shear induced by the sliding of basal ice, but if the sliding is a uniform motion in the downflow direction, we would expect the deposition and erosion rates to be in equilibrium. Other work specifically considers bed evolution in an effort to explain the evolution of subglacial bedforms [56], but we postpone including details of these processes for future work.

### (d) Mechanical coupling of the water and ice

It is necessary to prescribe a boundary condition at the interface between the ice and water in order to couple the subglacial hydraulic flow to the ice flow. This is done through a basal friction law, which relates the basal shear stress, ***τ***_b_, to the hydrology, through the general relationship
2.12
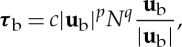

where **u**_b_=(*u*_b_,*v*_b_) is the sliding velocity of the ice, and *p* and *q* are commonly taken as 1/3 [3,7]. We furthermore take *c*=6.8×10^4^ Pa^2/3^ s^2/3^ m^−1/3^ so as to give a sliding speed of the order of 60 m yr^−1^ for typical driving stresses of 10^4^ Pa [57].

Coupling also occurs through feedbacks of the ice on the drainage system, in particular through the melting caused by basal friction and the creep closure of the ice over the water film. The prescription of the cooling rate, *q*_*T*_, in the melt rate expression (2.4) also depends on the ice velocity. To prescribe this, we first consider a simplified temperature equation. In a thermal boundary layer, the dominant balance is expected between heat advection and vertical diffusion
2.13
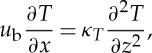

where *κ*_*T*_ is the thermal diffusivity of ice (and lateral heat advection is ignored because we expect *u*_b_≫*v*_b_ in the region of ice streams). With *T*=0 on *z*=*b* and 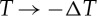 far from the bed, we obtain an error function as a similarity solution of (2.13), which yields the average basal heat flux in the form
2.14
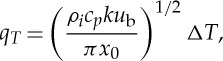

where the variable *x* has been replaced with the length scale *x*_0_. Constants are defined in table 1. This boundary layer approximation is appropriate if the reduced Péclet number 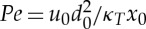 is large. For velocities *u*_0_∼100 m yr^−1^, length *x*_0_∼500 km and depth *d*_0_∼10^3^ m, *Pe*=5, and it is larger for ice-streaming speeds. The boundary layer approximation is not, therefore, a highly accurate approximation, but it probably gives a sufficiently accurate representation for the present purpose, and indicates correctly the increase of basal heat flux with basal velocity. Furthermore, the *q*_*T*_ boundary layer formula is inappropriate at large 
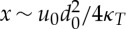
, when the temperature profile becomes more conductive, or for small *x* near divides where *u*_b_→0 and the *x* dependence becomes important. The Robin solution which includes vertical but not horizontal advection is often used here, but a better approach uses the von Mises transformation [58] which includes the Robin solution, and allows the similarity solution to approach divides by allowing for the *x* dependence of *u*_b_. However, we omit such subtleties here.

**Table 1.** Constants.

symbol	description	typical value
*ρ*_i_	ice density	917 kg m^−3^
*ρ*_w_	water density	1000 kg m^−3^
*Δρ*_wi_	density difference	83 kg m^−3^
*g*	gravity	9.81 m s^−2^
*η*_w_	water viscosity	1.8×10^−3^ Pa s
*η*_i_	ice viscosity	10^14^ Pa s
*L*	latent heat of water	3.3×10^5^ J kg^−1^
*n*	Glen’s flow law exponent	3
*c*_*p*_	specific heat capacity	2.1×10^3^ J kg^−1^ K^−1^
*ΔT*	surface cooling	20 K
*k*	thermal conductivity	2.1 W m^−1^ K^−1^
*c*	sliding law coefficient	6.8×10^4^ Pa^2/3^ s^2/3^ m^−1/3^
*p*	velocity exponent in sliding law	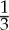
*q*	effective pressure exponent in sliding law	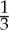
*G*	geothermal heat flux	60 mW m^−2^

From our simplified considerations, we therefore have that the sensible heat flux increases with *u*^1/2^_*b*_. The frictional heat, however, increases with *u*_b_, so, for large *u*_b_, *Γ* (2.4) is positive. It is possible for intermediate *u*_b_ that *Γ* could reach zero and then become negative; our model still applies until the water film thickness vanishes, at which point sliding law (2.12) would cease to apply and the model would need modification. However, in our simulations this never occurs because *G* and *τ* are sufficiently large to prevent refreezing.

## 3. Non-dimensionalization and reduction

Together with the equations for ice flow [48], the coupled system of equations includes the mass conservation of water equation (2.3) and the creep closure equation (2.6). Values of the dimensional constants are given in table 1. To couple the ice and water systems at the basal boundary, we have the sliding law (2.12).

### (a) Non-dimensionalization of the subglacial water equations

To make the subglacial water model dimensionless, we guess approximate scales for some variables based on observations/physical intuition, and then use these to derive characteristic scales for other variables, as given in table 2. We assume a characteristic value for the horizontal extent of the ice *x*_0_∼500 km based on length scales in ice-streaming regions, for example the Siple Coast [8]. We also define an effective pressure scale approximately 4×10^4^ Pa [59] and choose other scales by defining
3.1
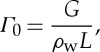

3.2
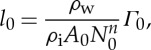

3.3
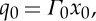

3.4
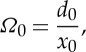

3.5
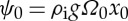

3.6
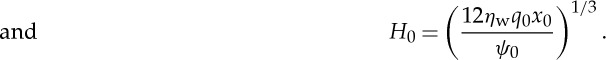

The ice flow scales are furthermore defined by
3.7
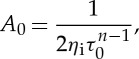

3.8
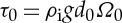

3.9
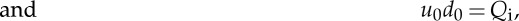

where the ice flux scale *Q*_i_ is either prescribed (from upstream supply) or related to the typical accumulation rate *a*_0_ by
3.10
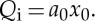

Equations (3.8) and (3.9), together with our prescribed sliding law (2.12), determine *τ*_0_, *u*_0_ and *d*_0_. However, as the sliding law is not well constrained, we will use observed values for these scales. Specifically, in the Siple Coast region of Antarctica, typical ice depths are 1000 m [60]. Given a velocity scale *u*_0_∼100 m yr^−1^, we take the accumulation rate scale as
3.11
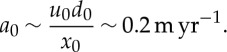

Finally, as the water flows on a much shorter time scale than the ice, we define two separate time scales
3.12
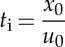

and
3.13
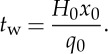

Table 2 gives values of these model variable scales.

**Table 2.** Scalings.

symbol	description	typical value
*x*_0_	horizontal length scale	500 km
*d*_0_	ice depth	1000 m
*Γ*_0_	melt rate scale	10^−10^ m s^−1^
*H*_0_	meltwater depth scale	3×10^−3^ m
*N*_0_	effective pressure scale	4×10^4^ Pa
*q*_0_	meltwater flux scale	5×10^−5^ m^2^ s^−1^
*u*_0_	ice velocity scale	100 m yr^−1^
*a*_0_	accumulation rate scale	0.2 m yr^−1^
*t*_i_	ice time scale	5000 years
*t*_w_	water time scale	1 year
*τ*_0_	basal stress scale	2×10^4^ Pa
*ψ*_0_	hydraulic potential scale	10^7^ Pa
*Ω*_0_	ice surface slope scale	2×10^−3^
*l*_0_	clast spacing	0.3 m
*A*_0_	Glen’s flow law rate scale	1.25×10^−23^ s^−1^ Pa^−3^
		=3 bar^−3^ yr^−1^

We non-dimensionalize all equations with the larger of the time scales, *t*_i_, and so introduce a non-dimensional parameter *δ*_*T*_=*t*_w_/*t*_i_. Using these scalings, the dimensionless form of the subglacial flow equations becomes
3.14
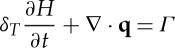

and
3.15
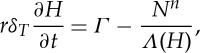

where
3.16
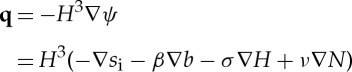

and
3.17
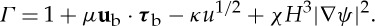

Dimensionless parameters are given in table 3 and *Λ*(*H*) is the same function as in (2.8), only now a function of the dimensionless water depth.

**Table 3.** Dimensionless parameters.

symbol	definition	typical value
*r*	*ρ*_i_/*ρ*_w_	0.9
*β*	*Δρ*_wi_/*ρ*_i_	0.1
*σ*	(*Δρ*_wi_*H*_0_)/(*ρ*_i_*Ω*_0_*x*_0_)	2×10^−7^
*ν*	*N*_0_/(*ρ*_i_*gΩ*_0_*x*_0_)	4×10^−3^
*χ*	(*q*_0_*ψ*_0_)/(*x*_0_*G*)	10^−2^
*μ*	(*τ*_0_*u*_0_)/*G*	1
*κ*	(*ρ*_i_*c*_*p*_*ku*_0_/*πx*_0_)^1/2^*ΔT*/*G*	0.27
*c*_0_	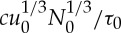	1.7
*λ*	(*τ*_0_*d*_0_)/(*η*_i_*u*_0_)	0.0625
*ε*	*d*_0_/*x*_0_	0.002
*δ*_*T*_	*t*_w_/*t*_i_	2×10^−4^

### (b) Simplification of the subglacial system

The parameters *σ*,*ν* and *χ* are all small. We thus neglect the terms of order *σ* and *χ*, representing gradients in water depth and melting due to meltwater dissipation, respectively, but retain the term in *ν*, as it represents a singular perturbation. From (3.15), we have
3.18
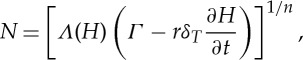

and the mass conservation of water equation (3.14) can then be written as an equation for *H*
3.19
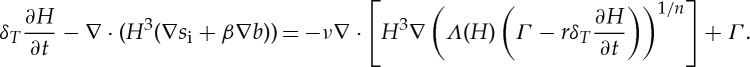

Furthermore, to allow consideration of the behaviour of the hydraulic system on its own, we temporarily assume that the ice has a constant, uniform surface slope
3.20
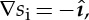

and zero basal slope, **∇***b*=**0**. Equation (3.19) then reduces to
3.21
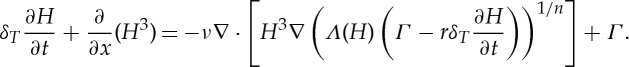



Given that *Λ*(*H*) is a decreasing function of *H*, the essential structure of the model can be described by
3.22
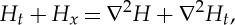

which is closely related to that studied by Benjamin *et al.* [61] as a model for long waves in shallow water; we may infer that the present equation is well posed.

### (c) Full set of governing non-dimensional equations

We now have a full set of non-dimensional governing equations for the water system. From Kyrke-Smith *et al.* [48], the non-dimensional mass conservation and force balance for the ice are given by
3.23
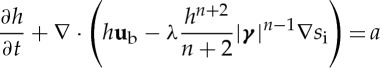

and
3.24
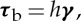

where
3.25
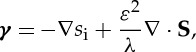

**S** is the resistive stress tensor [62,63],
3.26
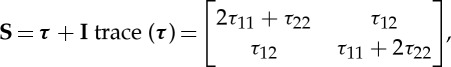

and *a* is the accumulation rate. ***τ*** is the deviatoric stress tensor, related to strain rate through Glen’s flow law for ice [8]. The full derivation and non-dimensionalization are provided in the appendix of a previous publication [48].

Together with the non-dimensional sliding law as the boundary condition at the water–ice interface,
3.27
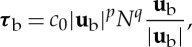

and the non-dimensional mass conservation of water equation (3.19), these equations provide a complete non-dimensionalized description of the ice–water system. Variable scales are in table 2 and descriptions of the non-dimensional parameters are in table 3.

## 4. Results for hydrology with constant ice

### (a) Hydraulic runaway

Hydraulic runaway is a physical phenomenon that models suggest may occur as a result of a positive feedback between ice velocity and melt rate [24]. As ice velocities increase, more meltwater is produced by frictional heating, which results in further ice–bed lubrication, and hence faster velocities. We consider whether the basal hydrology model presented here results in such behaviour.

The melting term, *Γ* in (3.21), depends on the ice flow. It therefore also has indirect dependence on the water depth, *H*, as the sliding velocity *u*_b_ depends on *H* through its dependence on *N*. The ice depth, *h*, evolves over a long time scale, *t*_i_, through the solution of (3.23) and (3.24). We are initially interested in changes in behaviour on a short time scale, so we assume that the ice is of constant depth, with constant surface slope (3.20). Taking the shallow-ice version of basal shear stress as an approximate sliding law, together with the water equations, we then have
4.1
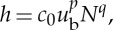

4.2
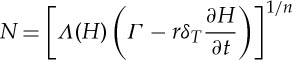

4.3
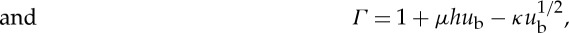

which gives three equations for the three unknowns, *u*_b_, *Γ* and *N*. These can be solved to give *Γ*=*Γ*(*H*,*H*_*t*_,*h*).

We seek to eliminate *u* and *N* from (4.1) to (4.3). Equation (4.2) gives us an expression for *N*(*Γ*,*H*) and substituting (4.2) into (4.1) we have an expression for *u*_b_(*Γ*,*H*) which is
4.4
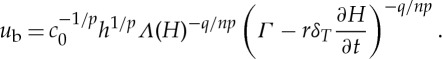

This can then be substituted into (4.3) so that we have *Γ*(*H*,*H*_*t*_,*h*).

**Figure 3.**
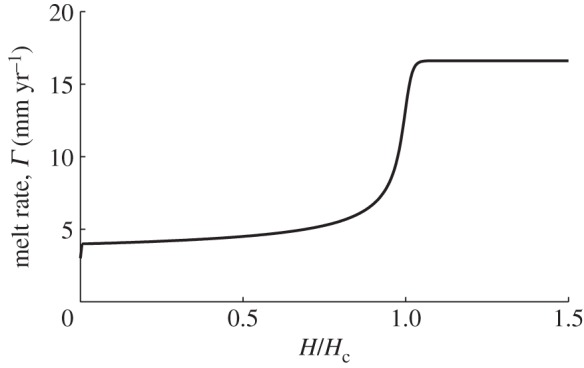
*Γ*(*H*) from (4.3) with (4.4), and *H*_*t*_=0, *h*=1, 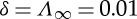 and other parameter values are as given in table 3.

In figure 3, we plot the quasi-static instance with ∂*H*/∂*t*≈0 and *Λ*(*H*) given by (2.11). As *H* increases, the melt rate also increases owing to an increase in frictional heating. This in turn comes about from an increase in basal velocity by (4.4). From this, it is evident that some kind of hydraulic runaway is inherent in the model, with melt rate increasing rapidly as 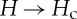. However, complete runaway is avoided as we do not allow the model to reach zero effective pressure in the subglacial system, which would correspond to no basal stress, and so allow the ice to increase in velocity without limit.

### (b) Solutions with a stable water film

We consider solutions of the decoupled water layer equation, to see its basic state behaviour. We take the ice to have constant surface slope and zero basal slope, as in the simplification given by equation (3.21). The coupling to the ice occurs through the melt rate term, which, from above, we know increases with both ice depth *h* and water layer depth *H*. We therefore here take an illustrative *Γ* function,
4.5
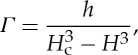

which results in an analytic steady-state solution to (3.21) at leading order. This steady-state solution is given by
4.6
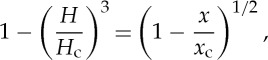

where
4.7
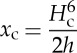

is the non-dimensional position downstream where *H* reaches *H*_c_. This corresponds to the point at which the bed becomes submerged and the thin film flow becomes a stream. If the bed is sufficiently rough (large *H*_c_) or the ice depth or flux is sufficiently small, then this point would be downstream of the grounding line, and the film flow can exist everywhere.

**Figure 4.**
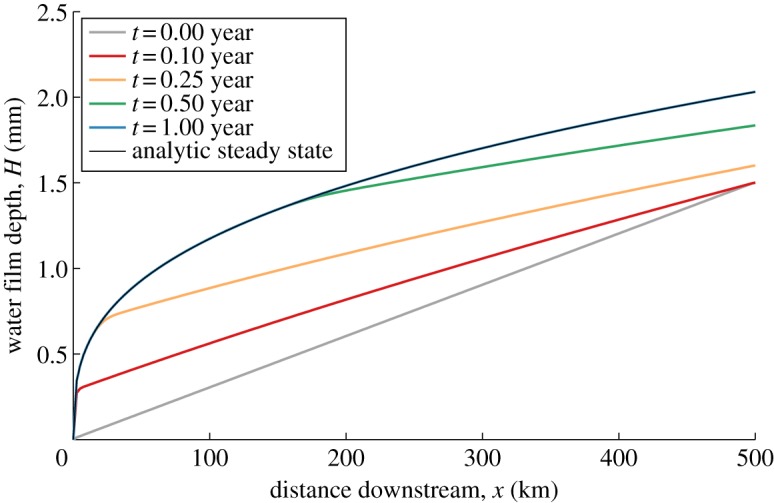
Evolution of a solution of the uncoupled water layer equation (3.21) with a melt rate given by (4.5). We take the ice to be of constant depth *h*=1 km and the critical water depth *H*_c_=4.5 mm. 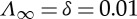 and other parameter values for the solution are given in table 3. The solution reaches the steady-state solution given by (4.6), plotted in black.

We ran a suite of simulations solving (3.21) with a melt rate of form (4.5). As expected, so long as *H*_c_>(2*h*)^1/6^ then the solutions evolve into the steady state given by (4.6) (e.g. figure 4). However, if *H*_c_<(2*h*)^1/6^ then the water depth reaches its critical value before the grounding line. The melt rate is no longer defined and an analytic solution does not exist. Our interest therefore now turns to the case where *H* may reach *H*_c_, by considering the fully coupled water–ice system.

## 5. Results for coupled hydrology and ice dynamics

Having formulated a description of the subglacial water layer, we now couple it with the ice to explore the combined behaviour of the system. The water film equation takes the same form, given by (3.19), and the ice flow equations are given by (3.23) and (3.24).

### (a) Model set-up and boundary conditions

We consider a 500×500 km domain with a uniform bedslope. The upstream end *x*=0 is a cold-temperate transition line with no water at the bed (*H*=0). The ice depth satisfies ∂*h*/∂*x*=0 across the boundary, and the inflow is at a constant velocity into the domain (*u*_b_=*u*_in_ at *x*=0). Free slip is also allowed along this inflow boundary (*τ*_12_=0). To avoid considering grounding-line behaviour, we treat *x*=1 as an open outflow boundary [26,64], where the upper surface is free to evolve and ∂*u*/∂*x*=0. Furthermore, a suitable condition for the water layer thickness, *H*, which also avoids the possibility of boundary layers, is ∂*H*/∂*x*=0. The domain is taken to be periodic in the *y*-direction. With these boundary conditions, we solve (3.21) coupled to the hybrid ice flow model outlined in §3*c*. The method for the numerical solution, using the Portable Extensible Toolkit for Scientific Computation [65–67], is described in previous work [48]. In that work, we used a heuristic triple-valued sliding law together with an equation to describe the relaxation time for the ice to adjust to changes at the bed; these have been replaced with a sliding law that directly depends on the subglacial conditions (3.27) and the physics-based water layer equation (3.19).

The simulations are initialized with a downstream ice flow that is uniform in the cross-flow direction, i.e. (*u*_b_,*v*_b_)=(*u*(*x*),0). The velocity field *u*(*x*) is computed from the *x*-component of equation (3.24) with (3.27). There is no water at the bed and the ice has a constant uniform thickness, *h*=1.5. A constant uniform accumulation rate is applied over the domain. Average accumulation rates in the Siple Coast region of Antarctica are approximately 140 kg m^−2^ yr^−1^ [68], and so we take *a*∼140 kg m^−2^ yr^−1^/917 kg m^−3^=0.15 m yr^−1^ as a suitable value. We expect the accumulation rate to be an important parameter in governing the flow behaviour, but do not present a detailed parameter study of the system in this paper; here, we discuss results that illustrate the basic flow regimes that occur as a result of a realistic accumulation rate applied over the domain.

### (b) Flow regimes

#### (i) Laterally uniform water and ice flow

**Figure 5.**
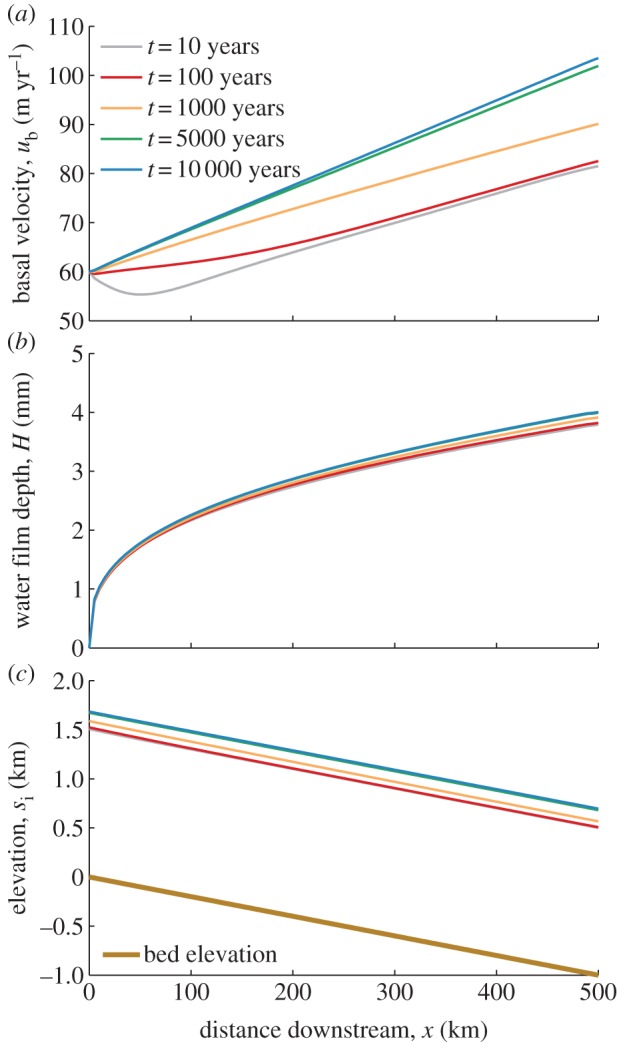
(*a*–*c*) Downstream centre-line profiles of basal velocity, water film depth and surface elevation plotted at five different times for the case where the water layer depth does not reach the critical value. Uniform accumulation of magnitude 0.15 m yr^−1^ is applied over the ice sheet and *H*_c_= 6 mm. There is an inflow of 60 m yr^−1^ into the domain. 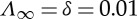 and other parameter values are given in table 3. The profiles remain uniform across the domain, hence we plot only a downstream profile.

We first consider the case where the water film does not get thick enough to submerge all surrounding clasts (i.e. the film depth remains less than the critical depth *H*_c_). If this is the case, the coupled system evolves into a stable steady state. This is illustrated in figure 5, where the critical water depth is *H*_c_=2, corresponding to 6 mm.

The water film evolves rapidly into a quasi-steady-state, similar to that seen in §4*b*, when the water layer is considered alone with a prescribed melt rate. Meanwhile, the ice depth and basal velocities also evolve into a steady state over a longer time scale. Ice depth remains uniform, increasing by approximately 200 m over the time of the simulation run. This is due to the downstream ice velocity initially being insufficient to balance the incoming ice flux and accumulation. However, the downstream ice velocity increases and over approximately 5000 years evolves to put the system in equilibrium. The velocity profile becomes a monotonically increasing function downstream. Over this time, the downstream profile of the water layer remains very similar to the initial profile it reaches within the first year. It does increase in depth by approximately 0.25 mm downstream owing to increases in ice velocity and ice depth that cause a larger melt rate from frictional heating. The water layer rapidly adjusts for this as the melt rate increase occurs.

Overall the model is well behaved, and the coupled behaviour is straightforward for the case where there is insufficient meltwater at the bed to submerge all supporting clasts.

#### (ii) Laterally unstable stream flow

Our interest now turns to the case where the amount of meltwater is sufficient to locally drown all supporting clasts; we are interested in what effect the decrease in support provided by the bed will have on the ice. Analysis in §4 suggested that melt rate will increase rapidly as the water depth reaches its critical value. In that analysis, we assumed that the ice was stationary as we were only interested in behaviour over a short time scale. As a result of that assumption, there is nothing to prevent water depth increasing indefinitely, in turn also allowing the ice velocity to increase (because there is little support at the bed). However, there is a long time-scale feedback that was not taken into account; over this longer time scale the ice depth decreases as a result of increased outflow from the domain. This causes the basal stress, and thus *Γ*, to decrease. The water depth will start to decrease as a result, therefore decreasing the ice velocity and the total ice flux. This cycle could then repeat once the water depth builds up again to reach the critical depth. In a narrow, confined flow, we might expect this to lead to cyclic surging behaviour (similar to [69]). However, in a wide flow, a lateral instability that leads to ice streaming occurs; we illustrate this with numerical solutions to the governing equations.

**Figure 6.**
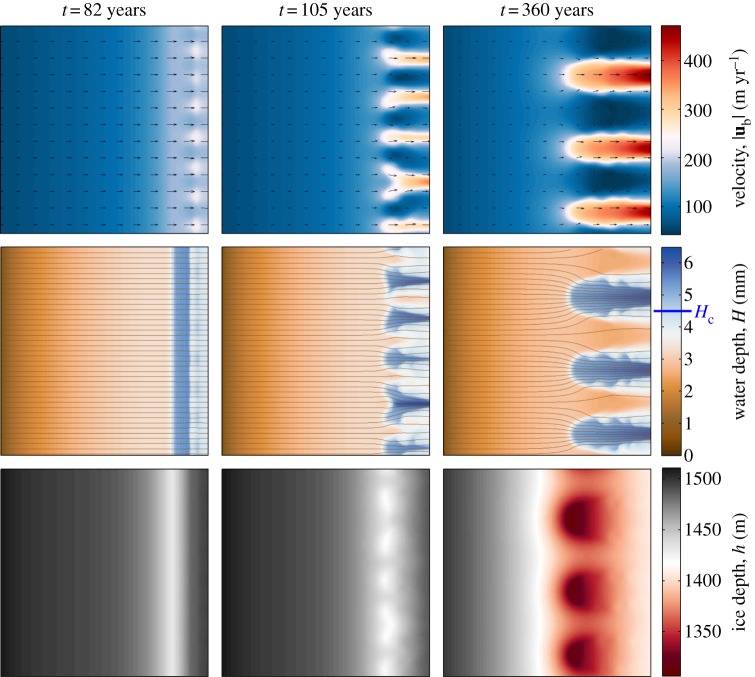
Velocity, water film depth and ice depth fields at three distinct times for a simulation run with *H*_c_=4.5 mm. Velocity vectors overlie the velocity plots and streamlines for the water flow overlie the water depth plots. Uniform accumulation of magnitude 0.15 m yr^−1^ is applied over the ice sheet. There is an inflow of 60 m yr^−1^ into the domain. 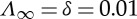 and other parameter values are given in table 3.

Figure 6 shows maps of basal velocity, water depth and surface elevation at three different times for a simulation run with *H*_c_=1.5, corresponding to a critical water depth of 4.5 mm. It is evident that this small change in the critical water depth results in dramatic changes in the behaviour of both the water layer and the ice, when compared with the results of the previous section. Initially, the flow is stable and uniform cross-stream, but, as more water is generated at the bed, *H* approaches *H*_c_ and an instability emerges, as shown in the plots at *t*=82 years. There is a sudden increase in water depth near the boundary, which results in an increase in ice velocity, because the ice can slip more easily over the bed. A consequential surface lowering of the ice is also evident in the ice depth field. A non-uniformity in the water depth and the ice velocity develops across the domain at this time, as a result of lateral instability (there has been no imposed perturbation to the system). Patches of faster ice flow form, corresponding to the positioning of the discrete areas of deeper water. There is a coarsening of this instability as the system evolves to create distinct stream-like features with a longer wavelength than the initial instability (*t*=105 years). The streamlines of the water flow show that water from upstream is drawn towards these water-rich areas, resulting in a positive feedback. These unstable features propagate up the domain, and evolve to create pronounced ice streams, flowing at hundreds of metres per year and of width approximately 60 km (*t*=360 years). The values are of the same order of magnitude as observed velocity and width values of ice streams in Antarctica [8], and in other numerical studies [38,70]. A discussion of the stress balances that are necessary to maintain ice-stream flow for different basal conditions is in previous work [48] and demonstrates the importance of membrane mechanics in determining ice-stream width. Again, the channelizing of water towards these deep-water patches is evident from the streamlines of water flow. There are depressions in surface elevation at the onset region of the ice streams, as a result of the rapid increase in discharge from the region. This is consistent with some observations [71]. At this point, the simulation has reached a quasi-steady state. The distinct streams do not grow further. There are, however, periodic wave-like features at the edge of the ice streams that propagate along the boundaries (particularly clear in the plots of water depth at 360 years and in the movie of the velocity field provided in the electronic supplementary material). These could be the result of travelling wave solutions that exist for the water layer equation (3.19), although this is purely speculative at present.

**Figure 7.**
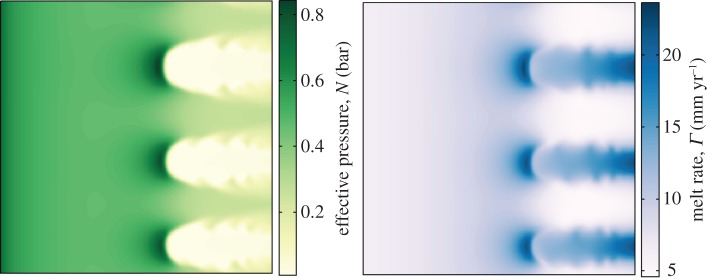
Effective pressure and melt rate fields plotted for the simulation shown in figure 6 at *t*=360 years.

Plots of effective pressure and melt rate at *t*=360 years are shown in figure 7. The fine-scale structures at the edge of the ice streams are evident in these fields as well. As would be expected from the relationship between *N* and *H* (equation (4.2)), we see that the areas of low effective pressure correspond directly to where there is a lot of meltwater. Low effective pressure furthermore allows the ice to move more rapidly because there is little resistance provided by the bed in these areas (from the sliding law (3.27)).

Moreover, the melt rate field illustrates the positive feedback that occurs with more melt being produced underneath the ice streams owing to frictional heating from the rapid velocities. The melt rate is, in fact, the greatest at the onset zone of the ice streams. This is due to the large driving stress here from the gradient in surface elevation, combined with a higher effective pressure, and therefore higher basal stress, which results in a larger melt rate from frictional dissipation. There is therefore enhanced melting at the onset zone—a feedback that will help to maintain ice-stream flow.

## 6. Discussion

### (a) Relationship to multi-valued flux laws

We have seen in the previous section that, under certain parameter combinations, ice flow can become laterally unstable; this leads to two distinct modes of flow—fast ice streams and slow-moving ice. This is similar to the behaviour seen in numerical models that use a triple-valued sliding law as a basal boundary condition [26,48]. Can the streaming results from the coupled ice–hydrology model be understood in terms of an emergent triple-valued sliding law, as with the more simplistic model of Fowler & Johnson [25]?

The water film evolves on a much faster time scale than the ice. As it is the ice flux we are interested in here, we assume now that the water film is in equilibrium, i.e. we take
6.1
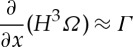

and
6.2
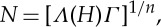

where ∂*s*_i_/∂*x*=−*Ω* is the ice surface slope, a constant in the downflow direction (we also assume there is no cross-flow variation in *H* or *Ω*). This is in contrast to the analysis of the water film in §4*a* when we were interested in the behaviour of the water film over a short time scale and so assumed the ice was in equilibrium.

Assuming that the melt rate is constant with *x* in this steady-state water case, and integrating (6.1) over *x* gives
6.3
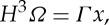

under the assumption that *H*=0 at *x*=0, i.e. the inflow boundary is taken to be a divide or a cold-temperature transition line.

We can write the non-dimensionalized sliding law (3.27) as
6.4
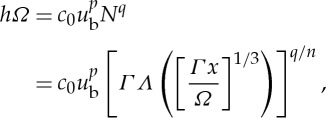

where
6.5
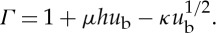



This gives a relationship between ice depth, *h*, and basal ice velocity, *u*_b_, for ice overlying a water film which is in equilibrium.

Using *Λ*(*H*) as described by (2.11), we have from (6.4)
6.6
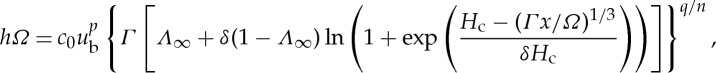

with *Γ* once again given by (6.5).

**Figure 8.**
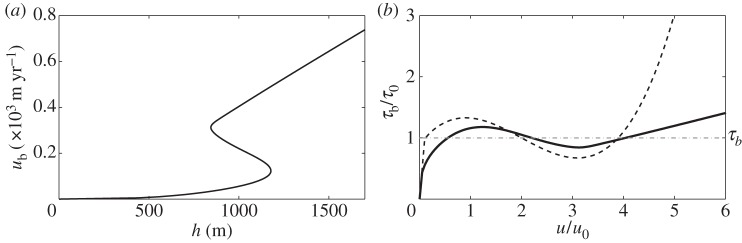
(*a*) Plot of one-dimensional ice velocity versus ice depth from (6.6) with non-dimensional parameter values *H*_c_=1.5, *p*=*q*=1/3, *x*=1, *Ω*=1.0, 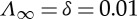, *μ*=1.0, *κ*=0 and *c*_0_=1.7. (*b*) The corresponding non-dimensional velocity against basal stress plotted alongside a rescaled triple-valued sliding law from Sayag & Tziperman [26] (dashed line).

We solve this algebraic relationship between *h* and *u*_b_ [72] and plot the solution in figure 8*a* with the same parameter values that produced the ice-streaming behaviour in §5*b*(ii). In this case, the relationship between ice depth and ice velocity is multi-valued, suggesting that distinct velocity states can occur at the same ice thickness, *h*. In figure 8*b*, we plot this multi-valued relationship against a rescaled version of the triple-valued sliding law derived in Sayag & Tziperman [26]. In their work, the sliding law is obtained analytically from a simple one-dimensional force balance, based on the cross-flow structure of an ice stream; it has been used for subsequent analysis of ice-stream behaviour by [48,73]. We see that the sliding law that arises from a basic coupling of our hydrology model and the ice flow exhibits very similar properties to the triple-valued function from Sayag & Tziperman [26]. As a result, when solving our coupled model, similar behaviour arises to that seen when using a triple-valued sliding law of the form presented in [26] as the basal boundary condition.

### (b) Parameter dependence

**Figure 9.**
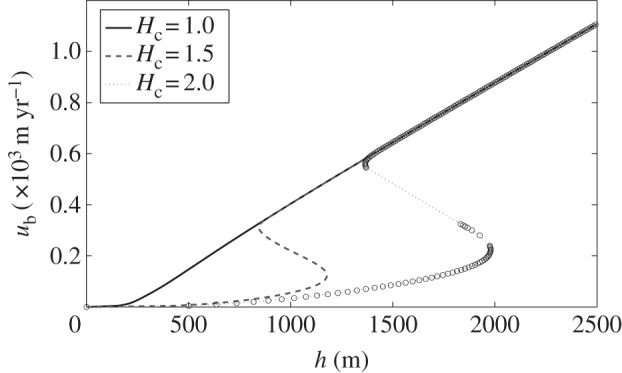
Plot of one-dimensional ice velocity versus ice depth from (6.6) with non-dimensional parameter values *p*=*q*=1/3, *x*=1, *Ω*=1.0, 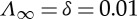, *μ*=1.0, *κ*=0 and *c*_0_=1.7. Plots for three different non-dimensional values of *H*_c_ are given, corresponding to 3, 4.5 and 6 mm. For *H*_c_=2 (6 mm) some solutions are imaginary and we therefore only plot the real solutions (circles), joined together by the dotted grey line.

The analysis above allows us to investigate what parameters are critical in governing multi-valued flux behaviour. Throughout the paper, we have used 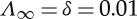 and have shown in figure 8 that this results in a multi-valued flux law when *H*_c_=1.5 (corresponding to 4.5 mm). We were motivated to plot this as we observed multi-valued behaviour from numerical simulations run with this parameter combination. The dependence of this behaviour on *H*_c_ is of interest; in figure 9, we therefore plot the relationship between ice depth and ice velocity for three different values of *H*_c_ with 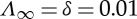. We see that while for very small *H*_c_ the relationship is not multi-valued, as *H*_c_ increases and the function becomes multi-valued the effect of further increasing *H*_c_ is that the turning points of the function occur at larger values of *h* and *u*_b_. This is because more meltwater is required for the water film depth to come close to the critical depth; a larger ice depth results in a larger driving stress that in turn causes more frictional heating. For larger values of *H*_c_ (e.g. figure 5), it is necessary to have thicker ice if there is to be sufficient meltwater to initiate the instability. This is in contrast to the behaviour we would expect if *H*_c_ were reduced too low; the flux law is no longer multi-valued, so we would not expect multiple velocity states to coexist in steady state, however large the driving stress.

**Figure 10.**
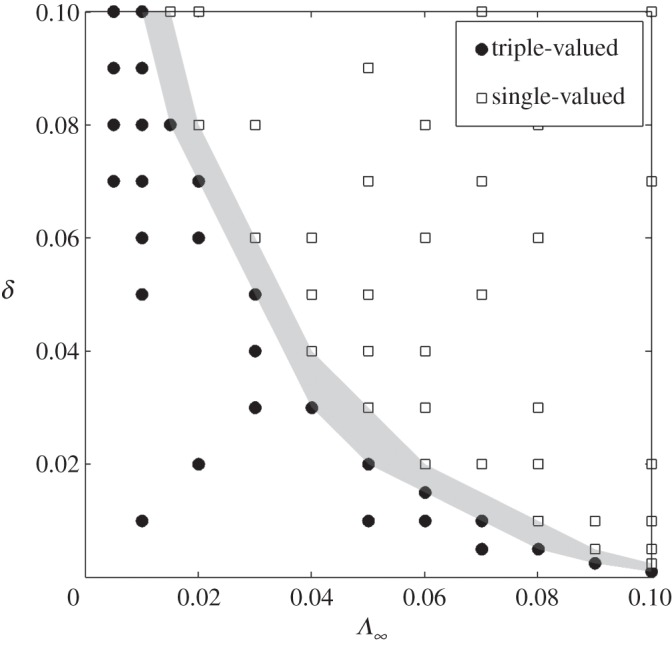
A diagram showing discrete regions where the ice depth versus ice velocity relationship is multi-valued and where it is single-valued, depending on the values of 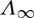 and *δ*. *H*_c_=4.5 mm as for the simulations shown in figure 8.

As well as considering variations in *H*_c_, in figure 10 we plot a phase diagram showing the distinct regions of 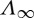
–*δ* parameter space where the relationship is triple-valued and single-valued for the case with *H*_c_=1.5. It is clear that if both 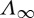 and *δ* are sufficiently small then the relationship (6.6) is multi-valued and there is therefore the potential for different velocity states to coexist.

## 7. Summary and conclusion

The theory developed in §2 provides a simplified model of subglacial water flow at the base of an ice sheet. We assume the meltwater flows in a Weertman–Creyts–Schoof style rough-bedded film [42,51] and show that this results in hydraulic runaway of the water film when the depth of the water layer reaches some critical depth *H*_c_ at which all supporting clasts are submerged. In reality, a water sheet with depth greater than *H*_c_ is an unstable configuration [52] and we would expect the water film to form discrete streams incised into the sediment; we present a simplified representation of this idea motivated by the fact that while locally there might be areas of zero effective pressure, at a larger scale (that is still sub-grid scale) there will be regions of finite support between water streams.

Numerical solutions of the fully coupled ice–water system result in distinct flow regimes dependent on whether the water depth reaches the critical depth within the domain. Under the model set-up considered in this paper, the system evolves into a uniform steady state if the bed is sufficiently rough (i.e. *H*_c_ sufficiently large) that all the melt can flow in the thin film configuration. However, reducing the critical water depth (so that *H* reaches *H*_c_) results in the ice flow becoming laterally unstable and non-uniform in the cross-flow direction; ice-stream-like features develop.

A basic analysis of the coupled system suggests that the hydrological system can result in a triple-valued flux relationship for the ice. It has previously been shown that a triple-valued sliding law is associated with ice-stream behaviour and such multi-valued flux laws have therefore been used to investigate ice-stream dynamics [26,48,73]. By showing that this coupling between subglacial hydrology and the ice can result in similar multi-valued flux behaviour, this work helps to contextualize analyses of ice-stream flow that result from using a triple-valued sliding law at the bed. Given that the sliding law is multi-valued, however, does not enforce instability in the system for all simulations; it still requires the ice to get sufficiently thick and the water layer to be sufficiently deep. Furthermore, some parameter combinations (we specifically consider variations of 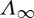, *δ* and *H*_c_ in this paper) do not result in triple-valued flux behaviour at all. In these cases, under no circumstances would we expect a lateral instability in the system to occur.

While this paper presents theory and intriguing results associated with a new coupling between physically reasonable models of ice and hydrology, a more detailed investigation of the coupled model behaviour is needed. Future work will provide a more comprehensive parameter study, as well as analysing in detail the stress balances that occur across emergent ice streams, in a bid to explain their spacing. Furthermore, an important limitation of the current model is that it neglects energy conservation. The model instead assumes that the bed is always at the melting point. While this might not be an unfair assumption in the region of ice streams, other work with thermodynamically coupled ice dynamics has shown that fast flow may begin owing to purely thermomechanical feedbacks [70,74,75]. It is interesting to observe from these studies that similar ice-stream widths are obtained both in thermal theories and in the hydraulic theory presented here. We expect that this is due to the role of membrane mechanics in resisting ice-stream flow, as discussed in our previous work investigating the necessary stress balances to maintain ice-stream flow [48]. It will be important in the future to consider a detailed model with energy conservation included, to explore the result of combined thermal and hydraulic feedbacks in a bid to further our understanding of ice-stream dynamics.

## Funding statement

This work was supported by the Natural Environment Research Council (NE/I528485/1). R.F.K. is grateful to the Leverhulme Trust for its support. A.C.F. acknowledges the support of the Mathematics Applications Consortium for Science and Industry (www.macsi.ul.ie) funded by the Science Foundation Ireland grant no. 12/1A/1683.
